# Clinical Holistic Medicine: the “New Medicine”, the Multiparadigmatic Physician, and the Medical Record

**DOI:** 10.1100/tsw.2004.25

**Published:** 2004-05-11

**Authors:** Søren Ventegodt, Mohammed Morad, Joav Merrick

**Affiliations:** ^1^ The Quality of Life Research Center, Teglgårdstræde 4-8, DK-1452 Copenhagen K, Denmark; ^2^ Division of Community Health, Ben Gurion University, Beer-Sheva, Israel; ^3^ National Institute of Child Health and Human Development, Office of the Medical Director, Division for Mental Retardation, Ministry of Social Affairs, Jerusalem and Zusman Child Development Center, Division of Pediatrics, Ben Gurion University, Beer-Sheva, Israel

**Keywords:** quality of life, QOL, philosophy, human development, holistic medicine, public health, medical chart, Denmark, Israel

## Abstract

The modern physician is often multiparadigmatic as he serves many different types of people in many different existential circumstances. The physician basically often has three, very different sets of technologies or “toolboxes” at his disposal, derived from three different medical paradigms: classical, manual medicine; biomedicine; and holistic or consciousness-oriented medicine. For lack of a better term, we have called the extended medical science — integrating these three different paradigms and their three strands of tools and methods — the “new medicine”. The excellent physician, mastering the “new medicine”, uses the most efficient way to help every patient, giving him or her exactly what is needed under the circumstances. The excellent physician will choose the right paradigm(s) for the person, the illness, or the situation, and will use the case record to keep track of all the subjective and objective factors and events involved in the process of healing through time. The case or medical record has the following purposes: *A. Reflection*: To keep track of facts, to provide an overview, to encourage causal analysis, to support research and learning, and to reveal mistakes easily. *B. Communication*: To communicate with the patient with a printout of the case record to create trust and help the patient to remember all assignments and exercises. *C. Evidence and safety*: To provide evidence and safety for the patient or to be used in case of legal questions. *D. Self-discipline*: To encourage discipline, as a good case record is basically honest, sober, brief, and sticks to the point. It forces the physician to make an effort to be more diligent and careful than a busy day usually allows.The intention of the case or medical record is ethical: to be sure that you, as a physician, give the best possible treatment to your patient. It helps you to reflect deeply, communicate efficiently, provide evidence and safety, and back your self-discipline, never to be carried away by the high speed of modern-day clinical work to give less than the optimal treatment. The patient's life, now and in the future, is in the palm of your hand, and to assume this huge responsibility, the physician must be anxious and careful about the quality of the medical record. Much too often, the essence of the session is nowhere to be found in the case record, so most of the generated value is lost between consultations.

## INTRODUCTION

All medical work is based on the intention of doing good, either improving health, quality of life (QOL), or the ability to function — or a combination. Independent of the good intention of the physician, medical work is always bound to some medical theory or a frame of interpretation. Hence the different paradigms[1] that give a number of different perceptions, hypotheses, diagnoses, actions, and reactions (comp. How we construct our consciousness in general and our reality[2,3]). The process of healing is, as life itself, often fairly complicated. The course of the disease, the healing process, personal development, learning, and coping in connection with a disease is often highly individual.

The modern physician is often multiparadigmatic as he serves many different types of people in many different existential circumstances. He basically has three, very different sets of technologies or "toolboxes" at his disposal, derived from three different medical paradigms:

- **Classical, manual medicine**, where the hands — used with the best and most humane intentions — constitute the main tools. It dates back to Hippocrates and Greek antiquity[4,5].
- **Biomedicine**, which came into widespread use around 1950, born paradigmatically along by the discovery of penicillin, where biomedicine focuses on body chemistry and physiology[6].
- **Holistic medicine or consciousness-oriented medicine**, which is a new and increasingly popular trend with many family physicians in the western world. It draws on a variety of healing processes, philosophies, and systems, taken in the original or modified forms from the premodern cultures. The most important thinkers influencing holistic medicine in northern Europe today are great physicians and philosophers like Jung[7,8], Maslow[9], Antonovsky[10,11], Frankl[12], Fromm[13], Goldman[14,15], Sartre[16], Kierkegaard[17], and Allardt[18]. The holistic approach focuses on the person as a whole, and this wholeness or soul or total existence is thought to be able to heal from its very totality — becoming "whole again", if the wholeness is lost partly or completely[19,20,21,22,23,24,25,26,27,28]

Depending on the perspective, or paradigm, very different things might happen to the patient when treated by the physician, and the signs and symptoms of development or progress of health and disease is interpreted very differently. If you go to a homeopathic doctor, which is fairly common in Germany for example, it is seen as a good sign if the treatment makes you feel worse for a while[29,30,31,32], but if you consult a biomedical doctor, then medicine is expected to make you feel better almost at once. If you consult a holistic doctor working according to the holistic process theory and the life mission theory[7,27,28], you would normally expect a very different path, being more happy and resourceful, and even when occasionally confronting painful old traumas, becoming more happy and resourceful. The reason for this is that the earliest existential wounds normally are the toughest to overcome, but the more resources you have, the more severe wounds on your soul, the more you will manage to confront and heal (Fig. 1).

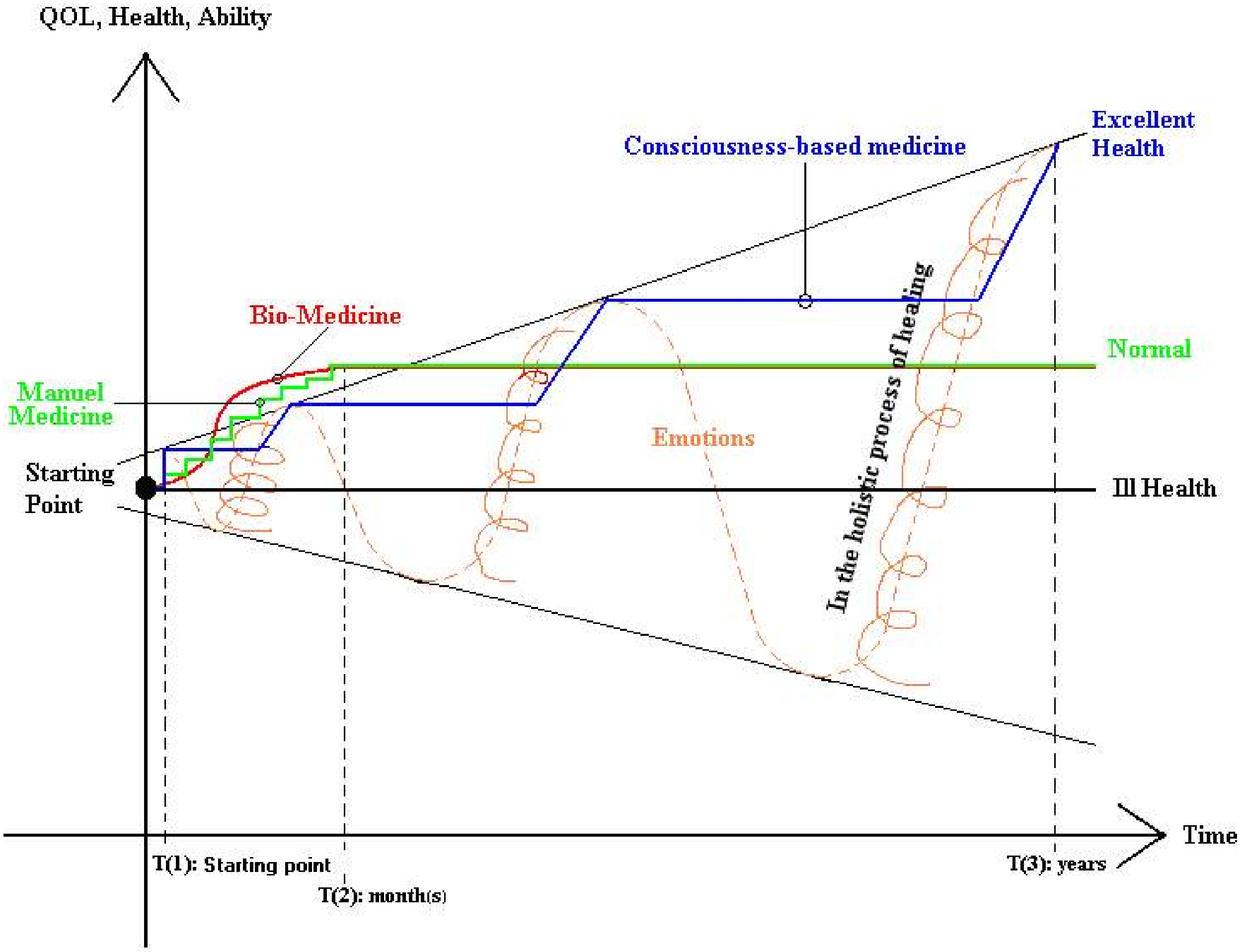

**FIGURE 1.** The common course of the healing process, within the three discussed paradigms. Where manual medicine often gives an immediate improvement of health, biomedicine takes a while, and holistic medicine has phases of holistic healing (often hours, marked coiled), phases of recreation (weeks to months), and prolonged phases of learning and personal growth (typically years). Manual medicine takes care of the body here and now. Biomedicine takes care of biochemical and physiological disturbances, often of long duration, if the disease is not cured but becomes chronic. Holistic medicine engages the patient in a process of existential healing and personal growth, often for life.

For lack of a better term, we have called the extended medical science — which integrates these three different paradigms and their three strands of tools and methods — the “new medicine”.

## CLINICAL HOLISTIC MEDICINE IS BASED ON THE LIFE MISSION THEORY AND THE HOLISTIC PROCESS THEORY OF HEALING

The life mission theory[19,20,21,22,23,24] states that everybody has a purpose of life, or a huge talent. Happiness comes from living this purpose and succeeding in expressing the core talent in your life. To do this, it is important to develop as a person into what is known as the natural condition, a condition where you know yourself and use all your efforts to achieve what is most important for you. The holistic process theory of healing[27,28] and the related QOL theories[33,34,35] state that the return to the natural state of being is possible whenever the person gets the resources needed for existential healing. According to the theory, the holding resources needed are awareness, respect, care, acknowledgment, and acceptance with support and processing of feeling, understanding, and letting go of negative attitudes and beliefs. The precondition for holistic healing to take place is trust with the intention that healing takes place.

Existential healing is not a local healing of any kind, but a healing of the wholeness of the person, making him/her much more resourceful, loving, and knowledgeable of him/herself, his/her own needs and wishes. In letting go of negative attitudes and beliefs, the person returns to a more responsible existential position and an improved QOL. The philosophical change of the person healing is often a change towards preferring difficult problems and challenges, instead of avoiding difficulties in life[36,37,38,39, 40,41,42,43,44]. The person who becomes happier and more resourceful often also becomes more healthy, more talented, and more able to function[45,46,47].

## CONSCIOUSNESS-BASED MEDICINE

In the search for the best way to make a new medical clinical practice and to serve the new type of patients we now see in our western society (the critical and knowledgeable patient or the patient focused on personal or spiritual development), we have worked with three different approaches to the new medicine:

- **Approach a. QOL as medicine**: Focusing on human feelings and emotions, we have combined biomedicine with a number of complementary therapies, like Rosen body work, classical Chinese acupuncture, and gestalt psychotherapy. We have called this holistic approach “quality of life as medicine”[45,46,47]. The combined treatment has the intention of inducing existential healing[27,28], and encompasses three phases popularly described as “feel, understand, and let go”: *feel* the blockages in body and mind, behind your health problems and symptoms; *understand* the life-denying conclusions you reached then which created them; and *let go* of these decisions once you are ready to assume responsibility and be your true, responsible self again. The team of physicians and alternative therapist complementing each other’s work under medical supervision could be the most efficient way to induce existential healing, in spite of the differences in professional language, culture, and paradigm.
- **Approach b. Meaning of life as medicine:** Focusing on the purpose of life, meaning of life, life mission, and talent[19,20,21,22,23,24]. Focusing on the hidden potentials, on the beauty and magnificence of the soul, and on the power of our existential choices, gives many patients faith and a fast healing progress. When the existential theories, the QOL philosophy and theories, and the QOL concepts are explained to the patients and internalized, patients gradually find themselves and return to a natural state of being, comparable in some aspects to the state in which they were born with a certain purpose of life and certain great talents to be used. The life mission theory simply states that denying your meaning in life leads you to illness, unhappiness, and poor performance, while recovering your purpose of life depends on finding and working for your purpose of life.
- **Approach c. Love as medicine**: Based on the concept of genuine human relationships and the power of unconditional love and acceptance, we have worked with the spiritual gift of love and the healing power of this in what we have designated an experimental, social utopia[47,48]. When patients belong to a small community with true companionship, contact, and emotional surplus, their way to recovery seems to be much shorter. The problem with social utopia is that it is very difficult to create and even more difficult to control. One of the preconditions seems to be that the participants do not have sex with each other, as this disturbs the possibility of intimacy in the group setting.

Although consciousness-based medicine supports individuals in their personal development, therapy and the patient-physician relationship can never replace a vibrant reality lived with those most important to them. It is the conquest of a good personal world to live in that can bring wholeness and healing. Quite simply, an individual can only realize the meaning and purpose of life in a social context. This purpose is what we are meant to be and with this gift we will be able to give to others. This can only happen most fully in intimate relationships, full of trust and love. A huge body of evidence has been collected on the connection between health and survival, and love and intimacy[49].

Many medical doctors seem to be unable to work with therapists that have not been trained scientifically, and many therapists do not like to be directed by a medical doctor, which makes an approach very difficult. Problems of this kind in the treatment team do not help the patients, as we have painfully experienced in our own clinical practice.

As human beings, we are limited to loving only a small percentage of our fellow men. This is an issue that naturally often grows to a larger fraction as we grow older, more wise, spacious, and containing, as we understand that love might be a leading concept in medicine — maybe even the strongest of the three concepts for inducing existential healing. Since such an approach for many seems unnatural, we are for all practical purposes left with the second approach in order for the modern physician to use the “new medicine”.

Interestingly, the three approaches above express to what degree the physician is willing to come close with the patient. This mirrors the intention of the physician towards his patient. In (a) the physician has the intent of helping the patient to heal, in (b) he/she has the intent of personally giving a gift to the patient from the bottom of his/her heart, and in (c) the physician has the intention to let the patient be a part of his/her life, in true appreciation of the magnificence of this unique soul in front of him/her. We believe that most physicians of our time who search their soul, will find that the intention of (b) is an appropriate ambition for their work. The physician who truly can give the holding[9,10] and processing in order to come close to his/her patients needs, will always be loved and respected by the patients.

## WHAT MAKES A PHYSICIAN EXCELLENT?

What will make a physician excellent are his/her good intentions, deep knowledge, and developed skills. In order to assist patients to a successful treatment and help the patients, the physician is only excellent when the good intentions result in the patient being adequately helped.

The patient is helped when one of the following two conditions is fulfilled:

1. The patient gets what he/she wants: QOL in some aspect or globally, health in some aspect or globally, or the ability to function in some aspect or globally — or a combination of these.
2. The physician gets what he/she wants: the broken leg healed or the disease treated or prevented.

So the situation is fairly complex, and much is depending on the physician choosing the right medical paradigm or toolbox. It is not easy to tell what a good medical treatment is unless:

1. You understand the paradigm chosen, and look at the patient from inside it.
2. You keep track of all the subjective, objective factors and events involved in the process of healing through time.
3. You have a valid way of testing the end result of the treatment.

All this is more or less complicated depending on the paradigm with the subjective paradigm the easiest to demonstrate[26]. This makes it surprisingly easy to make research and quality improvement in the holistic medical clinic, introducing existential healing according to the holistic process theory, and surprisingly difficult to document effect of the biomedical treatments, because of the objective approach. This later approach needs a difficult set-up with control groups in the Cochrane design to be valid.

## AN EXAMPLE OF THE THREE MEDICAL PARADIGMS AT WORK: LOW BACK PAIN

A patient comes to the physician with low back pain. If the physician uses manual medicine, he/she will examine the patient carefully to exclude the need of surgery. Then he/she will work with his/her hands on the patient, helping the patient to be more relaxed, less tense, and less in pain. Most fine body workers or chiropractors can remove a normal low back pain within an hour. When the cause in the body is understood and removed, the job is done. If the pain returns, so must the patient. If the patient gets a bad discus (a slipped disc) and a severe problem later with compression of the spinal nerves, it is not related to this treatment.

If the physician is working according to biomedicine, he/she will examine the patient carefully to exclude the need of surgery, and if the problem is not serious he/she will immobilize the patient, and use the painkillers necessary for this. He/she will talk about prevention, avoiding heavy lifting, or poor working postures. If the cause of the pain is understood this is fine, but mostly the low back pain has no objective cause and this is no obstacle for giving the treatment. When the patient is well again after the immobilization — it normally takes a couple of days — the job is done.

If the physician is working with consciousness-based medicine, he/she will examine the patient carefully to exclude the need of surgery. He/she will look for the cause of the illness in the patient's consciousness — difficult feelings repressed and placed in the longissimus thoraces muscles and other muscles. He/she will talk to the patient, give holding and processing, and inspire the patient to a more honest and joyful living. When the cause in the consciousness is understood and removed, and the pain is gone, the job is done.

It is not that any of these medical paradigms are better or worse than the other. The excellent physician mastering what we call the "new medicine" uses the most efficient way to help every patient, giving him or her exactly what is needed under the circumstances.

## WHAT IS THE PURPOSE OF THE CASE RECORD?

The physician is normally unable to make the quantitative research necessary to document the effect of his/her treatments, all though he/she can use the square curve paradigm or similar for quality improvement[26]. The best way of documenting the cure, noticing the paradigm in use, keeping track of all the subjective and objective events, and to see the final results is using the case record or chart. The case record has several general purposes[48], which include:

**A. Reflection**

- To keep track of facts. It documents the physician's actions and considerations, so that no patient is subjected to casual or careless treatment. Important issues are not forgotten. There is continuity of care.
- To provide an overview. The physician gains an important overview when writing down key information about patient and care.
- To encourage causal analysis. Very often, a physician may not realize that individual symptoms could be related. One symptom may lead to another, or, as is often the case, they may have a common underlying cause that the physician should be wise to look for, while reflecting on the patient's situation, when reading through the case record.
- To support research and learning. Detailed case records constitute unique tools of learning. When a physician writes down what is done and what is not done in each situation, it is possible to see afterwards what works and what does not work. In spite of all the learning in the world, each physician must draw his/her own conclusions and proceed by trial and error, and in this process the physician carries out a compulsory, qualitative research project with the case record at the center.
- To discover mistakes easily. Any mistakes made can often be discovered before the patient comes to any harm, if important therapeutic details are written down, such as the dosage of a drug, and the case record is read at the next visit.

**B. Communication**

- To communicate with the patient. Often we give the patient a printout of the case record so that he/she can remember any assignments or exercises, and can reflect at home on the potential causes of the disease and what he/she will have to do to get well.

**C. Evidence and safety**

- To provide evidence and safety for the patient. The case record protects the patient against errors in another important manner: under Danish law, a patient is entitled to a copy of his/her case record and to use it to consult another doctor who may then continue treatment and point out any irregularities.
- To be used in the physician's defense. The physician may need to document what he/she has done; for instance, if a patient lodges a complaint.

**D. Self-discipline and ethics**

- To encourage discipline and ethics. A good case record is basically honest, sober, brief, and to the point. Writing three lines following each session forces the physician to make an effort to be more diligent and careful than a busy day at the surgery usually allows.

So the intention with the case record is actually ethical: to be sure that you, as a physician, give the best possible treatment to your patient. It helps you as a doctor to reflect deeply, communicate efficiently, provide evidence and safety, and support your self-discipline, never to be carried away by the high speed of modern-day clinical work to give less than the optimal treatment. The patient’s life, now and in the future, lies in the palm of your hand, and to assume this huge responsibility, the physician must be anxious and careful about the quality of the case record. Much too often, the essence of the session is not to be found in the case record.

## SOME CASE EXAMPLES FROM FAMILY PRACTICE[48]

> **Female, aged 24 years with pneumonia:** She has suffered from chest tightness for 2 days. Auscultation of the lungs: Ronchi and "dense" sounds. Diagnosis: Pneumonia. Prescribe penicillin.

> **Male, aged 36 years with eye injury**: The patient was cutting stone with an angle grinder without wearing goggles and got what seems to be a glowing metal chip 1 × 0.3 mm in the left cornea at 7 o'clock seen from my position, outside the central vision. Chip removed with a needle point without any visible epithelial damage following anaesthesia. Eye pad. He was instructed to return in 3 days, if pain persisted. Prescribe Fusithalmic [fusidic acid] eye drops.

> **Female, aged 38 years with overweight**: Slimming plan. 131 kg. Presents fully motivated and has made the following plan: (1) swimming Tuesday + Thursday, (2) cycling to town and to bingo, (3) eating low-fat food, mostly crispbread with low-fat cheese. We talk about how diet should include many vegetables and little meat. She intends to live off her fat deposits and wants to lose 31 kg over the next 31 weeks to weigh 100 kg. This means lowering her daily calorie intake to two-thirds of current level. We talk about how to mentally overcome the sensation of hunger. Check-up every month.

The record or chart of the family physician is usually short and not like the usual chart in a hospital university department, where many students, physicians, and consultants have to be involved. The chart of the family physician is usually for his/her own utilization in order to keep track of patients and the treatment given. The three cases mentioned above are examples from a regular daily clinic of a busy family physician at work. Pneumonia is seen fairly often, especially in the winter period and straightforward to treat and the entry in the chart therefore very short and concise. Eye trauma is very important to treat at once and also important with follow-up in order to think about referral to a specialist. Obesity is many times a “mission impossible”, because if the lack of motivation from the patient and here follow-up and self-esteem are very important aspects of the treatment.

**Female, aged 56 years with growing old much too soon:**

1. Has slept on her side, pain corresponding to outside of left arm for 3 weeks. Loss of strength assessed as being of “protection – fixation type.” No sensory deficit, no affliction of feet or lower legs /to be followed up/.
2. Oedema (swelling) around the ankles. Prescribe Furix [furosemide] (a diuretic).
3. Patient requests a blood sample for gout, but I see no signs of this disorder in her, so there is no immediate indication for it. “If she doesn’t get it, her husband will tear the whole clinic apart.” She is informed in detail of the risk of prescribing too much medicine, if blood tests are not clinically justified and show false-positive results.
4. Productive morning cough for many months. Auscultation of the lungs: nothing abnormal discovered. No fever. May have slight bronchitis.
5. We talk about her everyday life, which is difficult; she becomes increasingly insecure. We talk about anxiety and menopause.
6. Headache almost daily. BP 130/90.

This patient has grown old 20 years too soon. She desperately wants to be examined, “since there must be a disease”, which the other physicians have overlooked and for which she can be treated. But on examination, there is no disease. A good physician usually knows intuitively whether or not people are seriously ill. To the best of our knowledge, this patient is not ill. We do not want to examine her for something we are certain she does not have — with the risk that the blood samples show a slight imbalance (one in twenty blood samples show a false-positive result). All the biomedicine in the world cannot save her. She has to save herself. Otherwise it will not happen.

So was this a good consultation? It depends very much on the consequences. Did the dialog inspire her to take more responsibility for her own life and health? As this is not likely, the answer seems to be in the negative, the consultation was less than excellent.

**Male, aged 13 years with psychosomatic abdominal pain**: Increasingly frequent, very severe abdominal pain for the past year, forcing the patient to unbutton his trousers and lie down until it passes. Examination: No blood in faeces, no vomiting or loss of appetite. Abdominal muscles very tender, especially at umbilical level on the right side. Otherwise soft, nontender, and without palpable masses. The abdominal pain can be provoked by manual stretching of the abdominal muscles. Socially: The patient’s parents have just separated. The patient says: “School is no good, they don’t teach you anything”, “mum tells me off a lot”, “she is stupid”, when she does that. He feels that it is a burden being the big brother of three sisters and responsible for them all. He cannot keep up at school and needs extra reading lessons; he is due to have some lessons after the holidays. The father feels that he is unable to get the school’s attention – it is like talking to a brick wall. And that reflects on the boy as well, Diagnosis: /Psychosomatic abdominal pain/. We agree that the father should discuss the situation with the mother and return with a plan, which we will go over together during a conversation.

Sometimes parents need to understand that their mutual conflicts can affect their children who can display their anxieties with psychosomatic symptoms. That is hard to acknowledge, because the last thing we want is to be bad parents, but a divorce need not always affect the child’s health, although that is very often the case. We have also seen several examples of women who become very abusive because they felt bad, while many men in a similar situation can become withdrawn and desert or neglect their child.

Probably the hardest thing for the child is the need to be loyal to both the father and the mother, and this can easily lead to a conflict that cannot be resolved. There is a deep psychological explanation for this need for two-sided loyalty, namely that each individual contains the two genders, represented by our parents. The quality of our future love life depends on how lovingly these two aspects of ourselves meet. Here in this case we see a different approach, the psychosocial approach characteristic of the consciousness-oriented medicine. The case record is good, because the psychosocial paradigm, the consequent actions, and the basic idea of the treatment are clear from the journal.

The next case shows the rhythm of the new medicine, integrating the three paradigms by alternating between them. Please note the nature of the exercises meant to raise the consciousness of the patient and help her assume responsibility:

> **Female, aged 28 years, who cannot function with boyfriend**: First QOL conversation. The patient suffers from lack of a boyfriend and a chronic feeling of being rejected – I would say that the feeling is: "He does not like me". Has been married, has two children, boy aged 8 and girl aged 5 years. The boyfriend before the husband broke her heart when, for the second time, he found someone else. She is "emotional", as though made for love, care, and sex, but she is blocked in this and is now unable to love. Her father regarded her as stupid and delightful. Her mother, whom the patient calls manipulative, found her irritating. The patient acted as a psychological mother for her own mother, and as a partner for her father. Examination: Tensions in the back and particularly in the abdomen, around the pelvis and the insides of the thighs. Cries when these tensions are contacted. There is a "pit in her abdomen".
>
> EXERCISE 1: Let go of negative decisions: "I'm no good" and "I'm irritating".
>
> EXERCISE 2: Patient is overweight — 10–20 kg — and eats in the evening in order not to feel. So: sit for 10 minutes daily in your emotional space and feel your emotion of being let down and rejected, insulted, fed up, and so on.
>
> EXERCISE 3: Find more negative decisions in their precise formulation and let go of them.
>
> PLAN: Rosen sessions (manual therapy) every 14 days, appointment with me in between if needed.
>
> Second QOL conversation. Her mother was always irritated with her. Since the patient was 3 years old, she has always been contrary, defiant, and stubborn. Did not want to show me her notes today. "I'm no good" is the basic problem, the patient says, and: "I'm not worth loving".
>
> EXERCISE 1. Describe all your advantages in wielding power.
>
> EXERCISE 2. Make lists of all your power games in relation to love, sex, and friendship, as well as work and motherhood.
>
> Third QOL conversation. The patient hands over a list of power games in relation to children and husband. It is clear that the patient wanted to rule and to control both her son and her ex-husband, when they were together. During the conversation, the understanding is crystallized in the sentence "I determine..." in the sense of....everything! Since she was 3 or 4, the patient has been "a sweet, warm-hearted and fair tyrant" in relation to those around her.
>
> EXERCISE 1: Let go of the sentence "I am the one who rules".
>
> EXERCISE 2: Accommodate your anger and other emotions, and be a pressure cooker for next time.

This kind of "I am the one who rules" decision, which guides the patient in her subconsciousness, is a very serious and destructive decision, which has been made at a time of extreme distress during childhood. When the patient finds it and lets go of it, all her energy and the whole quality of her personality will change radically. It is incomprehensible and quite alarming that our old decisions have such power over us that they destroy our life together throughout our lives. We create our life through our decisions. It is therefore vitally important to be clear about what decisions are at work here and now.

> Sixth QOL conversation. Condition: feeling of being rejected has disappeared. The feeling of being irritating has disappeared. Power games have disappeared; the patient spends time with her children in a far more caring and loving way. Very few conflicts with the children. “Yesterday they sprayed water all over the bathroom, and I didn’t even lose my temper. Previously I was not allowed to console my son or come close to him, now I can do that.” Finished.

This problem afflicts one in two modern people: we cannot make our life together work. Attempts are often made to solve problems in relationships by power. That is not nice. Resignation or break-ups and divorces are normally the result: submit or disappear! But before love finally dies out, the energy left in the love is often channelled into long and painful power struggles. The power games are generally based on earlier patterns of survival from childhood. When patients let go of their decisions about having to be in control and determine everything, they can then enter into a warm and rewarding relationship of love. Power games are highly destructive for love; fortunately most patients are willing to let go of the dark power games when their attention is drawn to them.

## DISCUSSION

In our wealthy society, almost every second individual suffers from chronic illness — a disease that is not cured in spite of modern medical treatment. One in five suffer from chronic pain[50,51], one in eight have a weight problem, one in ten an alcohol problem. Back pain, osteoarthritis, and rheumatoid arthritis, migraine, asthma, allergy, depression, hypertension, recurrent infections, personality disorders ... the list of chronic disorders in the modern Western society is long. This makes it clear that prevailing biomedicine, which seeks to treat almost everything with pharmacotherapy, is far from adequate.

Biomedical treatment is the right choice for many *acute* disorders and problems, but it is obviously not enough in chronic illness — or it would not have become chronic. Nor is biomedical treatment very useful against existential crises, burnout, social phobia, decreased libido, anxiety, age-related decline, dementia, or the other complaints that afflict so many people today.

What is wrong with biomedicine? As long as you only suffer from a variety of ailments and symptoms like mild pain, a slight allergy, mild depression, or sleeping problems the pills may well provide relief. Everybody can take pills, and in many cases they help us with what afflicts us, which is basically fine.

The difficulties with biomedicine begin at a later stage. Once we become really ill, which most people do in due course, the pills of biomedicine no longer suffice. So, once you become seriously ill, you will often become *chronically* ill. And what good is the physician and all his fine molecular medicine, the specially designed molecules of the pharmaceutical industry that cost billions to research, if the medicine cannot make us well again when we really need it? That is certainly one of the greatest problems of biomedicine.

One explanation of the shortcomings of biomedicine could be that many of our health-related problems are merely symptoms of the poor QOL and inadequate conduct of life that half of us experience: an unsatisfactory relationship without true love, a meaningless working life, no fulfilling leisure-time activities, and a family life without any real togetherness. This is the sort of life that will make us ill in due course.

At some point, we will need to assume more responsibility for our own life if we want to stay healthy. Here the physician may help to support and inspire the patient, increasing the patient's awareness of his or her options, hidden resources, and potentials. To this end, the physician needs an extended form of medical science that supplements our valuable knowledge of biochemistry with an insight in psychosomatics and consciousness.

## CONCLUSION

The “new medicine” that empowers the physician to help almost every patient that trusts in him, must include classical manual medicine, biomedicine, and holistic, consciousness-based medicine. Holistic means focusing on the patient’s soul and consciousness — his or her perception of life and the world and, in particular, his or her often considerable hidden resources. The actual guidance of the patients to make them assume responsibility for their own lives and mobilize their self-healing potential is one of the most important tasks of consciousness-based medicine, and of the modern day physician. Our consciousness carrying our purpose of life, our talents, and our love, may be the most characteristic feature of our personality as a whole, and enhanced awareness of our existence is the key to better existential choices, personal responsibility, and thereby a better life.

Encouraging their patients to assume more responsibility for their own health, QOL, and expression of skills and talents does not mean that there will be less to do for physicians. More than ever we need skilled physicians of good will and intention, who know their job: how to help people get well. As a well-educated and highly specialized expert in human healthcare, life, and well being, the physician has an important part to play in the society.

In the future society with many more conscious and enlightened people, knowing everything there is to know from the Internet, the medical doctor must be the carrier of wisdom, not only knowledge, if the physician intents to stay a valuable expert deserving the respect of his patients. The wise physician mastering the new medicine can help patients focusing on their efforts in defining their lives and providing a meaningful content. Personal development and existential healing seems to be important concepts for restoring the health, happiness, and ability to function of the people of our time. How the physician works with all the many aspects from the physical level to the spiritual, is best illustrated through the case record, where the stories about illness and recovery that they tell, can be used as teaching examples.

## ACKNOWLEDGMENTS

This study was supported by grants from IMK Almene Fond. The quality of life research was approved by the Copenhagen Scientific Ethical Committee under number (KF)V.100.2123/91.

## BIOSKETCHES

**Søren Ventegodt, MD,** is the Director of the Quality of Life Research Center in Copenhagen, Denmark. He is also responsible for a Research Clinic for Holistic Medicine in Copenhagen and is a popular speaker throughout Scandinavia. He has published numerous scientific or popular articles and a number of books on holistic medicine, quality of life, and quality of working life. His most important scientific contributions are the comprehensive SEQOL questionnaire, the very short QoL5 questionnaire, the integrated QOL theory, the holistic process theory, the life mission theory, and the Danish Quality of Life Research Survey, 1991–94 in cooperation with the University Hospital of Copenhagen and the late pediatric professor Bengt Zachau-Christiansen. E-mail: ventegodt@livskvalitet.org. Website: www.livskvalitet.org/

**Mohammed Morad, MD**, is Specialist in Family Medicine, Lecturer in Family Medicine at the National Institute of Child Health and Human Development, Division of Community Health, Ben Gurion University of the Negev and the Medical Director of a large area clinic in the city of Beer-Sheva. He has publications on Bedouin health, health aspects, spiritual health, and aging in persons with intellectual disability, and is a presenter on topics such as health policy and services for the disadvantaged at national and international conferences. E-mail: morad62@barak-online.net

**Joav Merrick, MD, DMSc,** is Professor of Child Health and Human Development affiliated with the Zusman Child Development Center, Division of Pediatrics and Community Health at the Ben Gurion University, Beer-Sheva, Israel; the Medical Director of the Division for Mental Retardation, Ministry of Social Affairs, Jerusalem; and the Founder and Director of the National Institute of Child Health and Human Development. He has numerous publications in the field of child and human development, rehabilitation, intellectual disability, disability, health, welfare, abuse, advocacy, quality of life, and prevention. Dr. Merrick received the Peter Sabroe Child Award for outstanding work on behalf of Danish Children in 1985 and the International LEGO-Prize (“The Children’s Nobel Prize”) for an extraordinary contribution towards improvement in child welfare and well being in 1987. E-mail: jmerrick@internet-zahav.net. Website: www.nichd-israel.com
